# Rhythmic Trafficking of TRPV2 in the Suprachiasmatic Nucleus is Regulated by Prokineticin 2 Signaling

**DOI:** 10.5334/jcr.ad

**Published:** 2015-04-01

**Authors:** Katherine J. Burton, Xiaohan Li, Jia-Da Li, Wang-Ping Hu, Qun-Yong Zhou

**Affiliations:** Department of Pharmacology, University of California, Irvine

**Keywords:** Prokineticin 2, TRPV2, Circadian Rhythm, Suprachiasmatic Neurons, Signaling

## Abstract

The mammalian circadian clock is composed of single-cell oscillators. Neurochemical and electrical signaling among these oscillators is important for the normal expression of circadian rhythms. Prokineticin 2 (PK2), encoding a cysteine-rich secreted protein, has been shown to be a critical signaling molecule for the regulation of circadian rhythms. PK2 expression in the suprachiasmatic nucleus (SCN) is highly rhythmic, peaking during the day and being essentially absent during the night. Mice with disrupted PK2 gene or its receptor PKR2 display greatly reduced rhythmicity of broad circadian parameters such as locomotor activity, body temperature and sleep/wake patterns. PK2 has been shown to increase the firing rate of SCN neurons, with unknown molecular mechanisms. Here we report that TRPV2, an ion channel belonging to the family of TRP, is co-expressed with PKR2 in the SCN neurons. Further, TRPV2 protein, but not TRPV2 mRNA, was shown to oscillate in the SCN in a PK2-dependent manner. Functional studies revealed that TRPV2 enhanced signaling of PKR2 in calcium mobilization or ion current conductance, likely via the increased trafficking of TRPV2 to the cell surface. Taken together, these results indicate that TRPV2 is likely part of the downstream signaling of PK2 in the regulation of the circadian rhythms.

## Introduction

The suprachiasmatic nucleus (SCN) of the hypothalamus houses the master circadian pacemaker in mammals [1]. At the molecular level, the circadian clock is controlled by positive and negative feedback loops involving transcription and translation of a handful of core clock genes [2]. The SCN is composed of many single-cell oscillators that, when synchronized, produce a coordinated circadian output [3,4,5]. One prominent feature of SCN neurons is the circadian oscillation of their firing rate, which peaks during the light phase [5]. Genetic studies in flies have revealed the critical role of firing rate oscillation in the generation of circadian rhythm [6]. Neurochemical and electrical signaling among SCN neurons is necessary for these individual cellular clocks to coordinate their activities and maintain robust oscillations [3,4,7,8,9].

Prokineticin 2 (PK2), encoding a cysteine-rich secreted protein, has been shown to be a critical signaling molecules for SCN neurons [10,11]. PK2 expression in the SCN is highly rhythmic, peaking during subjective day and being essentially absent during night hours [10]. Mice with the disrupted PK2 gene or its receptor PKR2 display greatly reduced circadian rhythmicity of locomotor activity, core body temperature, sleep/wake patterns, and other circadian parameters [12,13,14]. In terms of likely cellular mechanism of PK2 in the regulation of circadian rhythm, PK2 has been shown to increase the firing rate of SCN neurons [15]. The underlying molecular mechanisms of how PK2 signaling links to the increased excitability of PKR2 receptor-expressing neurons, including SCN neurons, are still unclear [16,17]. As previous findings have indicated that TRPV1, an ion channel belonging to the family of TRP, is a downstream signaling mediator of PKR2 in the activation of dorsal root ganglia neurons [18], we reasoned that a family member of this large TRP ion channels could play a role in PK2-enhanced electric activity of SCN neurons.

## Methods

### In situ hybridization and colocalization

Mice were anesthetized with xylazine and ketamine and transcardially perfused with PBS, followed by 4% paraformaldehyde in PBS. The brains were removed, post-fixed in 4% paraformaldehyde overnight, and cryoprotected with 20% sucrose in 0.1 M phosphate buffer (PB, pH 7.4) for 48 hr. The brains were subsequently frozen in isopentane and 16–20 micrometer coronal sections were cut using a cryostat. Antisense riboprobes containing the 3’ UTR of mouse PKR2 (GenBank accession number AF487279; residues 1147–2211) were generated by T7 RNA polymerase (sense probes were generated with SP6 RNA polymerase). Riboprobes was labeled with digoxigenin-UTP (DIG-UTP) (Roche) and diluted l to 50 in hybridization solution (50% formaldehyde, 10% dextran sulfate, 0.02% Ficoll, 0.02% polyvinylpyrolidone, 0.02% BSA, 500 µg/ml tRNA, 0.3M NaCl, 10mM Tris, pH 8.0, 1mM EDTA). For the hybridization, sections were sequentially hydrated in 1x PBS (phosphate buffered saline), 0.6% H_2_O_2_ in 1x PBS for 30 minutes, 1.2% H_2_O_2_ in 1x PBS for 1 hr, and 0.6% H_2_O_2_ in 1x PBS for 30 minutes. Tissue sections were fixed with 4% paraformaldehyde for 15 minutes, followed by three 1x PBS washes. Sections were pretreated with proteinase K (10 µg/ml) and pre-hybridized with the hybridization solution without probe for 1 hr at room temperature. Hybridized with the riboprobe, the sections were incubated at 60°C for 18 hours, followed by RNAase (20 µg/ml) digestion, decreasing salinity washes and a 60 minute high stringency (68°C) wash. Sections were then washed with 1x PBS and 0.2% Triton-X100 (PBST) twice, followed by a blocking solution (PBST, 3% BSA, and 5% horse serum) at room temperature for 1 hr. Sections were incubated with an anti-DIG antibody conjugated with horseradish peroxidase (Perkin Elmer) in blocking solution overnight at 4°C and washed with PBST. DIG labeled cells were revealed with the TSA Plus Fluorescence Kit from Perkin Elmer as described in the instruction manual. Sections were viewed with fluorescence microscopy prior to the immunohistochemistry. Sections were incubated with rabbit anti-TRPV2 antibody (1:1000, Santa Cruz Biotechology) for 24 hr at 4°C. After washes with PBST, the sections were incubated with the secondary antibody conjugated with anti-Rabbit-Cy3 (1:500, Jackson ImmunoResearch) for 2 hrs at room temperature. After washes with PBST, DAPI was added and sections were dried and cover-slipped. Confocal images were capitulated with Bio-Rad MRC 1024 confocal laser microscope. The use of Z-stack imaging was used to demonstrate co-localization. High resolution images were grayscaled and density was quantified using ImageJ 1.35s (NIH, USA). All procedures have been approved by IACUC committee of UC Irvine.

### Generation of stable CHO cells line expressing PKR2-apoprotein and TRPV2

Chinese hamster ovary (CHO) cells stably expressing PKR2 photoprotein aequorin (PKR2-aq) were grown in alpha MEM Earl’s Salts (Invitrogen, Carlsbad, CA), containing 10% fetal bovine serum, 100 µg/ml streptomycin, 100 U/ml penicillin, G418 (100 µg/ml), and Zeocin (100 µg/ml). Human transient receptor cation channel, subtype V, member 2 (TRPV2) cDNA (ATCC) in pIRESpuro was transfected into PKR2-aq using lipofectAMINE manufactures protocol (Invitrogen, Carlsbad, CA). Clones of cells stably expressing PKR2 and TRPV2 were selected under 5µg/ml puromycin in a 5% CO_2_ incubator at 37°C. Colonies were tested using the LB12 Sirius Luminometer (Berthold) as described [19].

### Ca^2+^ mobilization assay

Activation of TRPV2 has been shown to be induced by serum and thus all cellular experiments were pretreated with 6 hours serum-starvation [20]. Cells were serum starved at 37°C in B27 supplemented alpha MEM (Invitrogen, Carlsbad, CA) for 4 hours and in reduced serum OptiMEM I (Invitrogen, Carlsbad, CA) with coelenterazine cp (8µM) for an additional 2 hours. Stable cell lines were then trypsinized, counted, centrifuged, and resuspended in calcium-free HBSS (Hanks’Balanced Salt Solution) plus 10mM HEPES (4-(2-hydroxyethyl)-1-piperazineethanesulfonic acid). The luminescence of both PKR2 and PKR2-aq:TRPV2 stable cell lines was measured with a Berthold Luminometer as follows.

After serum starvation, CHO cells were resuspended in Ca^2+^-free HBSS. Cell analysis occurred in a bioluminescence assay measuring changes in intracellular Ca^2+^ ([Ca^2+^]_i_) concentrations both with application of HBSS or PK2. CHO cells were subjected to PK2 for 10 mins, followed by serum-free defined media. Cells were fixed at 0, 10, 30, 60, 90, and 120 mins and immunostained using extracellular-recognizing TRPV2 antibody.

### Voltage-clamp electrophysiology in Xenopus oocytes

Linearized cDNA-containing vectors for PKR2 and TRPV2 were used to transcribe cRNA using mMessage mMachine reagents (Ambion, Austin, TX). Concentrations of cRNA were determined by spectrophotometric absorbance and by gel electrophoresis.

For oocyte isolation, sexually mature female *Xenopus laevis* were anaesthetized and lobes of ovaries were removed and stored at 16°C and gentamycin (Sigma). After oocytes were plucked and denuded by gentle vortexing with 1mg/ml collagenase (Worthington, Lakewood, NJ), healthy oocytes were injected with PKR2 cRNA or PKR2 and TRPV2 cRNAs.

*Xenopus laveis* oocytes were placed in a 35mm chamber with a bath solution containing 96mM NaCl and 2.5mM BaCl_2_. The cells were impaled with two glass electrodes filled with 3M KCl connected to a two-electrode voltage clamp TEV-200 amplifier and oscilloscope. Once impaled, oocytes were incubated in various concentrations of Ruthenium Red for 5 minutes (Alexis, San Diego, CA) and were followed by PK2. For each experiment, 50nM PK2 was added for 40 seconds. All recordings and images were generated using pClamp 9 Software Suite (Axon, Union City, CA).

### Statistical analyses

Data were analyzed using GraphPad Prism Software Version 5.0 (San Diego, CA). Two-way or one-way ANOVAs with Bonferroni post-hocs and unpaired t-test were used.

## Results

### Robust Expression of TRPV2 in the SCN and colocalization of TRPV2 with PKR2 in the SCN neurons

**Supplemental Figure S1:**
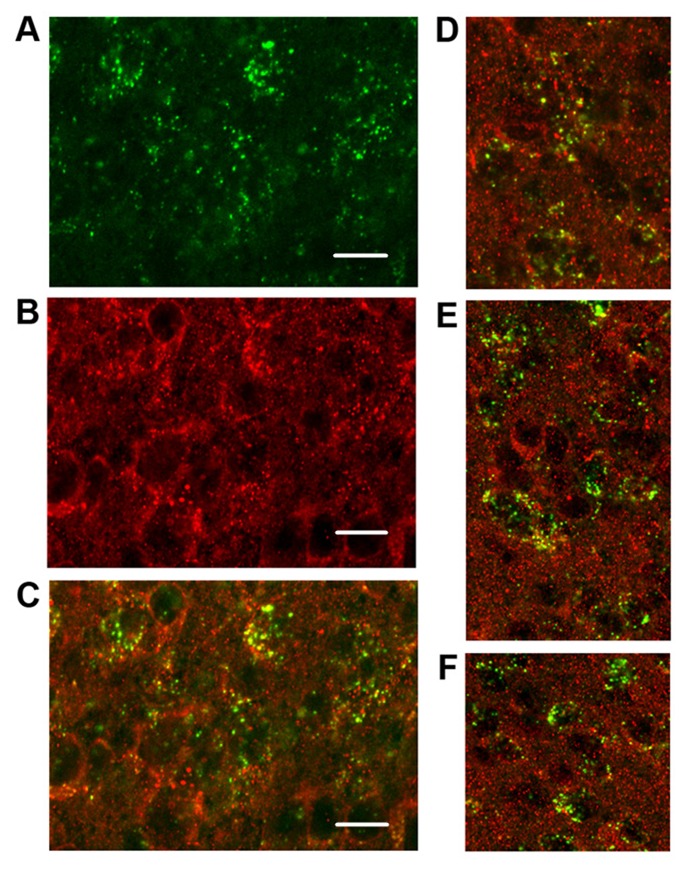
Screening of 33 Trp channels revealed expression of candidate TRP in the SCN of adult mouse brain. Autoradiograms show mRNA expression in the SCN of ***A***, PKR2, ***B***, TrpV2, ***C***, TrpC1, ***D***, TrpC2, ***E***, TrpC4, ***F***, TrpC5, ***G***, PKDILI-1, ***H***, TrpM7, ***I***, PKD1, ***J***, PKD2, ***K***, TrpV3.

**Figure 1:**
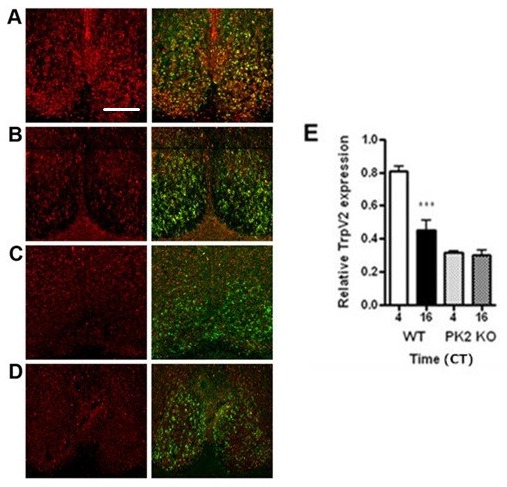
Colocalization of PKR2 mRNA and TrpV2 protein expression in SCN. **A**, PKR2 mRNA and **B**, TrpV2 protein in SCN neurons of WT mouse. **C**, Colocalization of TrpV2 and PKR2 in A and B. Scale bars=50 µm. **D-F**, Confocal images of colocalization in the SCN neurons, 100x magnification. TrpV2 protein (red); PKR2 mRNA (green).

We screened the expression of all 33 known Trp channels in the mouse SCN [21,22] by *in situ* hybridization. The majority of the 33 known Trp channels were not expressed in the SCN, with several of them, including TRPC1, C4 and C5, being lightly expressed in the SCN (Supp. Fig. S1). The only robust expressed TRP channel was TRPV2 (Supp. Fig. S1). In addition to its robust expression in the SCN, TRPV2 was also expressed in the several known SCN target areas, such as paraventricular and dorsal medial nuclei of the hypothalamus. As the expression pattern of TRPV2 and PKR2 in the SCN and several known SCN targets is quite similar, we thus investigated whether PKR2 and TRPV2 are co-expressed in the same SCN neuron with combined *in situ* hybridization and immunostaining. Fig. 1*C-F* shows that TRPV2 is indeed co-expressed with PKR2 in the SCN neurons.

### PK2-dependent oscillation of TRPV2 protein in SCN neurons

**Supplemental Figure S2:**
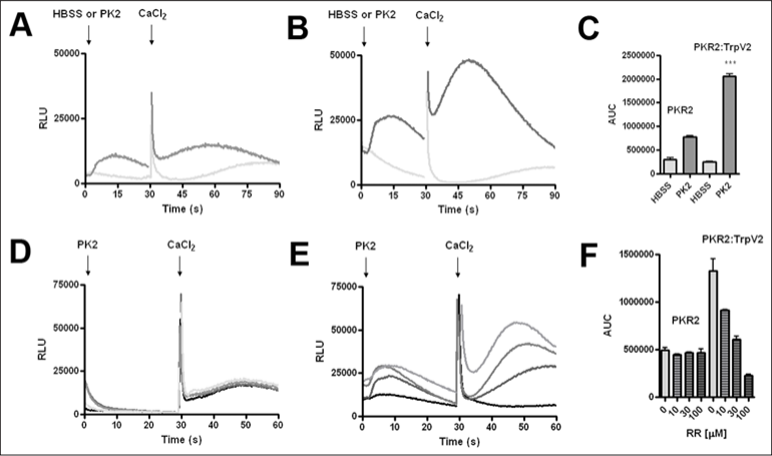
Absence of oscillation of TrpV2 mRNA in the SCN. *A*, PK2 mRNA oscillates over a 24 hr period in the SCN. *B*, TrpV2 mRNA remains unchanged over 24 hr period. *C*, Quantification by MCID analysis of PK2 and TrpV2 mRNA. Animals were subjected to constant darkness for 2 days. Each value is the mean of 4–6 animals. Solid squares, PK2; open squares, TrpV2. Scale bar=1 mm.

**Figure 2:**
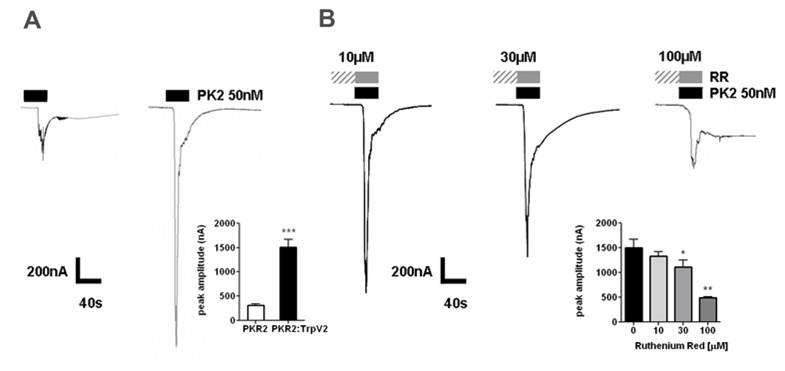
Oscillation of TrpV2 protein expression in SCN of WT and PK2 KO mice. A, Coexpression of TrpV2 protein and PKR2 mRNA in the SCN of WT mouse at CT4. TrpV2 protein expression is reduced in the SCN of WT mouse SCN at CT16 (B). TrpV2 level are similar in the SCN of PK2 KO mouse at CT4 (C), and CT16 (D). Images of left column are of TrpV2 protein (red) alone; images of right column are of TrpV2 protein and PKR2 mRNA (green). E, Graphical representation of TrpV2 protein level in the SCN of WT and PK2 KO mice at CT4 and CT16 ***p<0.0001. Scale bar=500 µm.

The circadian profile of TRPV2 expression in the SCN was then examined. Levels of TRPV2 mRNAs were observed to be non-oscillating in the SCN over a 24 hour period (Supp. Fig. S2). However, immunochemistry studies revealed a marked oscillation of TRPV2 protein expression in the SCN: TRPV2 protein level at CT4 was almost two fold higher than at CT16 (Fig. 2*B, E*). As PKR2 is coexpressed with TRPV2 in the SCN neurons, we next examined whether the circadian oscillation of TRPV2 is linked to PK2 signaling with PK2-deficient (PK2-/-) mice. As shown in Fig. 2, the oscillation of TRPV2 was absent in the SCN of PK2-/- mice: with the SCN at CT4 of PK2-/- mice displaying similarly low level as that of CT16 (Fig. 2*C-E*).

### Enhanced Ca^2+^ mobilization in PKR2-TRPV2 co-expressing cells

**Figure 3:**
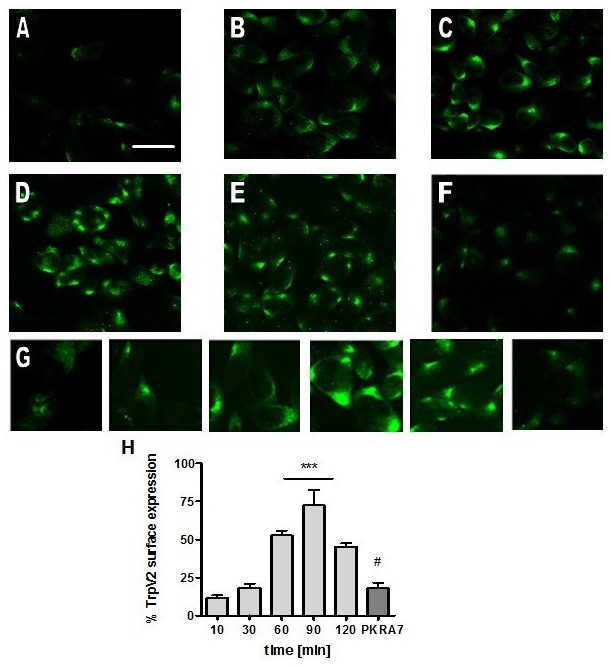
PK2-induced Ca2+ mobilization of PKR2:TrpV2- expressing CHO cells. ***A***, PKR2 and ***B***, PKR2:TrpV2 CHO cell Ca2+ mobilization in Ca2+ free HBSS. Initial injection at 1 sec of either HBSS and PK2 followed by 3 mM CaCl2 injection at 30 sec. ***C***, Graphical representation of area under the curve (AUC) for calcium peak, ***p<0.0001. ***D***, PKR2-expressing CHO were unaffected by 3 min pretreatment of increasing doses of ruthenium red (RR), Results are ± SEM from 5–6 separate experiments. ***E***, RR dose-dependently decreased the calcium mobilization inPKR2:TrpV2-expressing CHO cells. ***F***, Calcium mobilization was significantly increased by the expression of TrpV2, which was inhibited by RR.

To determine the functional interaction of PKR2 and TRPV2, we next examined Ca^2+^ mobilization in stable cell lines that co-express PKR2 and TRPV2 (PKR2:TRPV2). As shown in Figure 3*A-C*, the calcium mobilization signal was significantly increased in PKR2:TRPV2 cells, compared to parental PKR2 cells [19]. The modest mobilization of Ca^2+^ in the parental PKR2 CHO cell line can be attributed to calcium release from intracellular storage (Fig. 3*A* and 19). Furthermore, ruthenium red, a TRPV blocker, was shown to dose-dependently inhibit the calcium signaling in PKR2:TRPV2 cells (Fig. 3*E-F*). This decrease was due to blockade in TRPV2 channel activation, since [Ca^2+^]_i_ concentrations in the parental PKR2 cells were unaffected (Fig. 3*D, F*).

### TRPV2 increased membrane current through PKR2 activation in Xenopus oocytes

**Figure 4:**
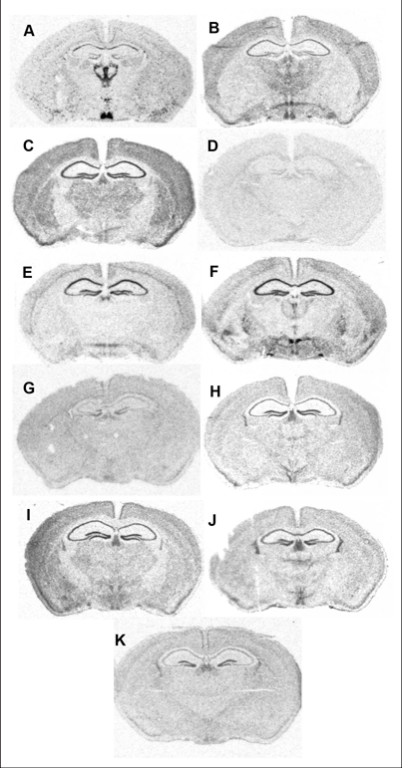
PK2-evoked current in oocytes that coexpress PKR2:TrpV2. ***A***, PK2 (50 nM)-evoked currents in PKR2- (left) and PKR2:TrpV2-expressing (right) oocytes. Graph inset displays the significance in peak amplitude, ***p<0.0001. PKR2 injected, n=15; PKR2:TrpV2 co-injection, n=11. ***B***, Oocytes expressing PKR2:TrpV2 were pretreated with RR (3 min). Graph inset shows the decrease in peak amplitude with increasing doses of RR. **p<0.01, *p<0.05; n=20.

We further used two-electrode voltage clamp electrophysiology to examine the functional interaction of TRPV2 and PKR2 in *Xenopus* oocytes. In oocytes co-injected with PKR2 and TRPV2 cRNAs or injected with PKR2 alone, exposure to PK2 evoked inward currents (Fig. 4*A*). Peak currents were about 5-fold higher in PKR2:TRPV2 injected oocytes compared with PKR2 alone (Fig. 4*A*, graph inset). Onset of currents occurred rapidly within ~5 secs in PKR2:TRPV2 oocytes with an aciculate profile. In contrast, with mean peak current amplitude of 400nA, PKR2-expressing oocytes had a slower PK2-induced onset of ~15 secs and displayed undulatory properties, typical of GPCR electrophysiological profiling [23]. Peaks returned to baseline more slowly in the PKR2-expressing oocytes and often displayed a prolonged oscillatory-IP3 patterning, also indicative of GPCR-driven signaling (Fig. 4*A*).

To verify that the functionality of the TRPV2 channel is under the regulation of PKR2, we further used ruthenium red to block TRPV2 channel activity. In oocytes expressing PKR2:TRPV2, ruthenium red dose-dependently attenuated PK2-evoked currents (Fig. 4*B*, graph inset). In contrast, ruthenium red did not alter current amplitude in oocytes expressing PKR2 alone. These studies indicated that TRPV2 increases oocyte membrane current through PKR2 activation.

### PKR2 activation increases TRPV2 expression at the cell surface

**Figure 5:**
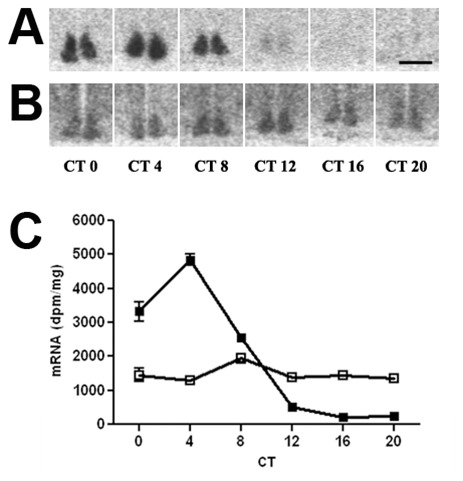
PK2-induced cell surface translocation and large cluster formation of TrpV2 in CHO cells. All cells were serum-starved and subsequently treated with PK2 or PK2 antagonist. Cells were then fixed at indicated time points and immunostained using a rabbit-anti-TrpV2 antibody. ***A***, no PK2 added, ***B***, 30 min, ***C***, 60 min, ***D***, 90 min, ***E***, 120 min. Pretreatment with ***F***, PKR antagonist (PKRA7). Scale bar=40 µm. ***G***, Higher magnification images (63x) of individual CHO cells. Left to right equates to images A-F. ***H***, Percentage of cells showing translocation of TrpV2 in response to PK2. ***p<0.001, #p<0.05.

**Supplemental Figure S3:**
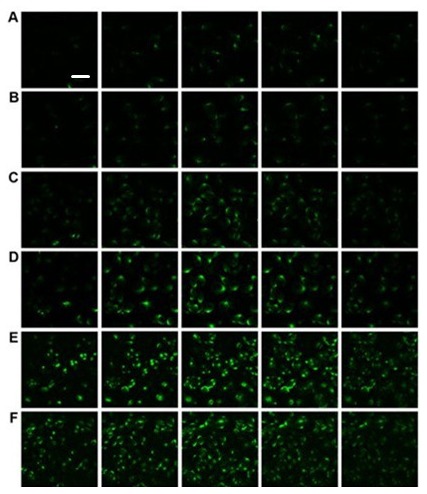
Z-stacking confocal imaging of TrpV2 translocation to surface membrane. ***A***, no PK2 treatment. CHO Cells were treated with PK2 for 10 min ***B***, 10 min, ***C***, 30 min, ***D***, 60 min, ***E***, 90 min, ***F***, 120 min. Note the apparent cell surface expression of TrpV2 in response to PK2 treatment. Cell surface expression of TrpV2 was detected by immunostaining with anti-TrpV2 antibody. Scale bar=40 µm.

The molecular mechanism for the functional activation of TRPV2 is still unclear, with one likely possibility being that the TRPV2 channels are constitutively active and their activity is regulated by trafficking to cell surface expression [20,24,25,26,27,28,29]. We thus set out to examine the trafficking properties of TRPV2 in response to the PKR2 activation. In absence of the PKR2 activation, TRPV2 protein is low at the cell surface (Fig. 5*A*). 30 mins after addition of PK2, TRPV2 was increased at the cellular membrane (Fig. 5*B*). TRPV2 expression increases more by 60 min, and peaks at about 90 min (Fig. 5*C, D*). Interestingly, robust expression levels of TRPV2 were observed in the formation of large clusters on the membrane, particularly around peak level (Fig. 5*D, H*). Confocal images of Z-stack (Supp. Fig. S3) clearly demonstrated the robust trafficking of TRPV2 to the cell surface membrane via the activation of PKR2. To further ascertain this trafficking to cell surface expression of TRPV2 was truly due to the activation of PKR2, we pretreated the cells with a selective PKR2 antagonist, PKRA7 [30,31]. Fig. 5*F, H* shows that PKRA7 blocked the cell surface expression of TRPV2 induced by PKR2 activation. Taken together, these results indicate that PKR2 activation increases the trafficking of TRPV2 to cell surface.

## Discussion

The coexpression of PKR2 and TRPV2 in the SCN neurons was demonstrated by combined in situ hybridization and immunostaining (Fig. 1). Further, TRPV2 protein, but not TRPV2 mRNA, was shown to oscillate in the SCN in a PK2-dependent fashion (Fig. 2). Functional studies indicated that the co-expression of TRPV2 enhanced signaling of PKR2 in calcium mobilization or ion current conductance, compared to cells or oocytes that express PKR2 alone (Figs. 3, 4). Morphological studies indicated that the enhancement of PKR2 signaling by TRPV2 is likely via the increased trafficking of TRPV2 to the cell surface (Fig. 5). Taken together, these results indicate that TRPV2 is likely part of the downstream signaling of PK2 in the regulation of circadian rhythms [13,14,15].

There exist several models that describe how TRPV2 channel activation occurs [32], with one possibility being that TRPV2 channels are constitutively active and their activity is regulated by trafficking to cell surface expression. It has been previously shown that TRPV2 trafficking to the plasma membrane surface is increased in response to the signaling of growth factor receptors, such as insulin-like growth factor-I (IGF-I) or other receptor [20,23,27,33]. It has been further shown that PI3 kinase is likely involved in the cell surface expression of TRPV2 induced by IGF1 [20]. In this report, we showed that PKR2 activation by PK2 in CHO cell induced the cell surface trafficking of TRPV2 (Fig. 5). It should be pointed out that PKR2 receptor activation has previously been shown to couple to the activation of PI3 kinase [34], and thus a similar signaling pathway may play the role of controlling TRPV2 trafficking to cell surface.

The oscillation of the electric firing rate of the SCN is known to be under the control of the circadian clock, but the exact molecular and cellular mechanism is still largely unknown [3,4,7,8,9,35,36]. Clock-controlled genes may directly encode ion channel [37] or regulate ionic conductance activity in the SCN neurons [35,36,38,39,40]. PK2, as the product of a clock-controlled gene, has been shown to be one of the signaling mediators for the normal expression of circadian rhythms [10,13,14,41]. With regard to the circadian rythmicity of the firing rate of the SCN neurons, PK2 has been shown to increase the firing rate of rat SCN neurons [15]. Our current results indicate that PK2 signaling increases the trafficking of TRPV2 to the cell surface. In the absence of PK2 signaling in PK2-deficient mice, the TRPV2 protein levels in SCN neurons are diminished during the subjective day and no longer oscillate between circadian day and night phases (Fig. 2). Thus, oscillating PK2 in the SCN may contribute to the circadian oscillation of SCN neuron firing rate by regulating the trafficking of TRPV2 to the cell surface of SCN neurons.
